# The Heating and Ventilating of the New Harvard Medical Schools

**Published:** 1906-04-28

**Authors:** 


### THE HEATING AND VENTILATING OF THE NEW HARVARD MEDICAL SCHOOLS.

At the new Harvard Medical Schools at Boston, which are in course of construction, and which consist of five large schools in separate blocks of three stories each, with a power-house occupying a sixth block, ventilation is provided by a combination of the plenum and the exhaust systems. There are ten fans, each 15 ft. in diameter, providing fresh air, and thirty-two fans, each 7 ft. in diameter, for exhausting the foul air. Heating is by the forced hot-water system, the water being heated at a central point by steam and forced to radiators in all the rooms, returning to the heaters after doing its work on the radiators. The air is filtered by passing through a number of fine canvas bags on its way to the fresh-air fans, the bags being taken down and cleaned when required. Heating is done partly in the neighbourhood of the fans and partly in each room, the latter being by means of the hot-water radiators mentioned. The standard temperature for the inside of buildings in America is 70° F., as against 60° with us, the difference being stated to be owing to the hygroscopic question. In America the air is much drier; consequently a higher temperature is required to provide a comfortable humid condition. Professor Woodbridge, who is an authority on the subject in America, has taken up the question of the effect of the dust that is deposited in the air-ducts. The Professor states that he has examined numerous samples of this dust, and has always found them perfectly harmless. His theory is that the large quantities of oxygen contained in the air passing through the ducts thoroughly purifies the dust.

